# Postoperative and long-term survival in relation to life-expectancy after pancreatic surgery in elderly patients (cohort study)

**DOI:** 10.1016/j.amsu.2021.102724

**Published:** 2021-08-15

**Authors:** S.K. Burgdorf, J.H. Storkholm, I.M. Chen, C.P. Hansen

**Affiliations:** a Department of Surgery Rigshospitalet, University of Copenhagen, Denmark; b Department of Oncology, Herlev Hospital, University of Copenhagen, Denmark

**Keywords:** Pancreatic cancer, Elderly patients, Life-expectancy, Pancreatic surgery

## Abstract

**Background:**

An evaluation of the outcome after pancreatic surgery with focus on post-operative and late survival in elderly patients was performed.

**Methods:**

The study included 1.556 patients from a single HBP unit operated from 1. January 2010 to 31. December 2019. Patients were divided into two cohorts, < 75 years (*n* = 1.296) and ≥75 years (*n* = 260). Post-operative outcome was evaluated in all patients and late outcome in patients with adenocarcinoma in the pancreas (*n* = 765) and the duodenum (*n* = 117). The follow-up of patients with benign disease and adenocarcinoma was 57.95 (12.1–132.7) and 39.85 (12.0–131.7) months, respectively.

**Results:**

Length of hospital-stay and surgical complications were not significantly different in the two cohorts, but in-hospital death was 1.1% (<75 years) and 3.5% (≥75 years) (*p* = 0.008). The median overall survival of adenocarcinoma was 29.7 (<75 years) and 24.3 months (≥75 years) (*p* = 0.3228) with a one, two, and five-years survival of 74.5%, 56.6% and 28.6% vs. 73.6%, 51.1%, and 25.5%. Median time to relapse (46.2% of patients <75 years and 40.5% of patients ≥75 years) was 9 (1 - 51) and 8 (1 - 78) months (p = 0.534), respectively. Adjuvant chemotherapy did not have impact on the survival of the old cohort. Patients who died during the observation period had lost 94% (<75 years) and 87% (≥75 years) of expected remnant life. Estimated years lost in the old cohort was 4.2 in males and 4.9 in females (*p* = 0.025)

**Conclusion:**

Elderly patients may undergo pancreatic surgery with a low mortality and for adenocarcinoma with an acceptable long-term survival.

## Highlights

• Surgery is the only potentially curable treatment to pancreatic cancer.
• Elderly patients may tolerate pancreatic surgery with low mortality.
• Radical pancreatic surgery improves long-term survival, also in elderly patients.
• Operability should be evaluated from morbidity and biological age.
• Pancreatic surgery in elderly should only be performed in high volume centers.

## 1 Introduction

Pancreatic cancer is mainly seen among elderly subjects as more than 85% of all patients are diagnosed after 60 years of age. Today, pancreatic cancer is the seventh leading cause of cancer death in both sexes worldwide [1,2], the fourth leading cause of cancer-related death in the United States and is expected to be the second in 2035. The incidence of pancreatic cancer is seen world-wide concurrently with the ageing population. With an increase of life expectance in the affluent part of the Western World, there will be an increased demand of therapy including surgical treatment in a population with an increased prevalence of co-morbidity [3].

Pancreatic surgery is complex due to complicated resections and reconstructions. With an intimate location to major vessels, radical surgery may require concomitant vascular resection. Pancreatic surgery in elder patients have earlier been avoided due to a high perioperative mortality which even in younger patients exceeded 20% in several low-volume centers. Although the postoperative mortality has decreased and today is less than 5% in high-volume centers, many surgeons are still reluctant to offer pancreatic surgery to the elderly. However, studies have shown an acceptable short-term mortality and morbidity after pancreatic operations in the elderly as well as a survival benefit in patients with pancreatic ductal adenocarcinoma (PDAC) [4,5]. Many data, however, are inconsistent and do not differentiate between the short-term outcome, which depends on patients’ preoperative health status and postoperative complications, and the long-term outcome, which depends on the pathology. Moreover, several large-scale studies include results from multiple centers or from national databases, and less from single centers. This may conceal the results from less experienced centers and give a skewed impression of the risks and benefits of pancreatic operations in old patients.

When offering an old patient pancreatic surgery for a malignant disease it is important to evaluate not only the probability that the patient may survive the operation, but also the relevance of an operation in relation to the patient's remnant life expectancy and other treatment options than surgery. This problem has not been addressed in most studies on pancreatic surgery in the elderly, in which the outcome after surgery was the end point.

In a cohort of patients from Copenhagen University Hospital Rigshospitalet we evaluated the short-term outcome after pancreatic surgery in patients aged 75 years and older compared to younger patients. Moreover, we investigated the long-term outcome after surgery for adenocarcinomas in the pancreas and duodenum to evaluate, if operation is justified in elderly patients with respect to life expectancy in an age-matched background population.

The endpoints of the study were the post-operative survival and surgical complications after major pancreatic surgery in all patients operated in the study period, and the long-term survival of patients operated for adenocarcinomas. These endpoints were set from the assumption that the postoperative mortality is related to age and comorbidity, while the long-term survival is influenced by the primary disease.

## 2 Methods

### 2.1 Study population

The study is a single-center study and includes all patients after pancreatic resection from 1. January 2010 to 31. December 2019. The hospital is a tertiary center for hepato-pancreato biliary surgery with a catchment area of around 2.5 mill people. In 2020, 241 pancreatic operations for malignant and premalignant diseases were carried out at the hospital.

Patients were divided into two cohorts, younger than 75 years (group A) and 75 years and older (group B). There are various definitions of old age but no agreement about the time, when a person is old [6]. WHO defines an elderly person in the Western world by the age of 65 years. In Denmark, the median age of patients with pancreatic malignancy lies in the mid-sixties, so an old patient in this respect had to be defined by a higher age. Our decision to use 75 years as the point of intersection was arbitrary. To study the postoperative morbidity and mortality, both cohorts were further divided into patients with either malignant or benign conditions.

### 2.2 Data collection

Data were collected from our prospectively maintained database of pancreatic operations, from the electronic hospital record systems Orbit and EPIC, the Danish National Pathology Data Registry, and from the National Register of Death. All Danish Nationals have a unique Central Person Registration number that enables searching of health data. Patients from the Faroe Islands and Greenland were excluded, as they are not recorded in the Danish death register. The follow-up period ended 31. December 2020 one year after the last patient was included, so all patients were followed for a minimum of one year or until death. No patients were lost to follow-up.

### 2.3 Patients

All patients underwent preoperative staging by diagnostic imaging, which included a triple-phase multidetector-row computed tomography and if needed supplementary magnetic resonance imaging, endoscopic ultrasonography, and positron emission tomography. All images were evaluated and thoroughly discussed with assessment of resectability at our multidisciplinary tumor conference in the presence of surgeons, oncologists, and dedicated radiologists.

Before operation, a pancreatic surgeon and an anesthesiologist evaluated the patient's operability in the out-patient clinic, and a dedicated counseling providing the patient with information and goals for recovery was given. Preoperative optimization of organ dysfunction was performed, and all patients with heart disease, earlier heart surgery or 80 years and older underwent preoperative cardiologic examination including echocardiography and, if necessary, interventional coronary angiography. Patients with pulmonary disease had pulmonary function test and echocardiography. Stenting of the biliary duct by endoscopic retrograde cholangiopancreatography (ERCP) or percutaneous transhepatic cholangiography (PTC) was performed in patients with jaundice if operation could not be performed in less than one week or if serum bilirubin exceeded 100 μmol/L to avoid renal toxicity. No patients had a WHO performance score exceeding 2.

### 2.4 Treatment

All surgical procedures were standardized. Pancreaticoduodenectomy and total pancreatectomy were performed with all anastomoses on the same jejunal loop without pylorus-sparing procedure. The pancreaticojejunostomy was performed either as a Blumgart (duct-to-mucosa) procedure or as an invaginated end-to-side anastomosis depending on the caliber of the pancreatic duct [7]. In case of porto-mesenteric vein resection venous reconstruction was performed with an end-to-end anastomosis, or less commonly, with a bridging graft, either the patient's umbilical vein or a necro-donor vein. Portal flow was ultrasonographically monitored during vascular surgery and postoperatively.

Patients with adenocarcinomas were followed until two years after the operation or until recurrence, in which case they were referred to the oncologic department. Follow-up with clinical assessment and clinical chemistry including serum cancer-associated antigen CA 19-9 was undertaken every three months the first postoperative year and every six months the second year or on demand. Since there is no present evidence that routine thoracoabdominal CT scan has an impact on survival this examination was only performed if recurrence was suspected, but on wide indications.

No patients received neoadjuvant therapy, but all fit patients with adenocarcinoma were offered postoperative oncologic evaluation regarding adjuvant therapy. Patients with PDAC, papillary carcinoma of the pancreato-biliary type and cholangiocarcinoma followed the ESPAC-3 protocol with adjuvant gemcitabine [8] until August 2016, where the ESPAC-4 protocol with gemcitabine and capecitabine was implemented [9]. From July 2018 combination therapy with FOLFIRINOX (folic acid, fluorouracil, irinotecan and oxaliplatin) was included in the adjuvant therapy for fit patients [10]. Due to the lack of evidence patients with duodenal carcinoma were only offered adjuvant therapy in cases of regional lymph node metastases and/or low differentiation of tumor, in which cases most patients were treated with FOLFOX (folic acid, fluorouracil, and oxaliplatin).

### 2.5 Guidelines

Resectability complied with the criteria and guidelines of the US National Comprehensive Cancer Network (NCCN) [11] and the European Society for Medical Oncology (ESMO) [12]. Preoperative risk assessment was recorded as medically treated comorbidities and scored according to the Charlson Age-Comorbidity. Grading of postoperative pancreatic fistulas followed the International Study Group of Pancreatic Fistula (ISGPS 2016) [13]. TNM staging followed the American Joint Committee on Cancer, eighth edition.

### 2.6 Outcomes

Relevant postoperative complications were recorded in the study and included leakage from the pancreatic, bile or gastrojejunal anastomosis, intraabdominal hemorrhage and abscess formation or other complications with severe or fatal outcome. Outcomes were defined as postoperative complications and mortality assessed during 30- and 90-days and overall survival (OS) defined as the time from surgery to death from any cause or censoring at time of last follow-up. Hospitalization was defined as postoperative stay until discharge. In-hospital mortality was defined as all deaths from time of admission until discharge. Cancer specific mortality was defined as death from adenocarcinoma after other causes were censured. Recorded years of life lost was defined as lost years compared to the expected remnant life of an age-matched standard population. The recorded years of life lost were the number of deprived years that could have been saved, if patient had not developed an event that had shortened life.

### 2.7 Ethics

The study is a descriptive study and was conducted in accordance with the principles stated in the Declaration of Helsinki. No approval was required according to the Danish National Health Board. The use of register data followed the General Data Protection Regulation of the European Union and was approved by the Danish Data Protection Agency (RH -2015-07, nr. 03616) and patients’ consent. The study was registered at clinicaltrials.gov under ID: NCT04893408.

### 2.8 Statistics

The study was reported according to the STROCSS guidelines [14]*.* Data are presented as median and range if not otherwise stated. Categorial data are presented as numbers or percentage and were analyzed with Fisher's exact test. Non-parametric continuous data between subgroups were analyzed with the Mann-Whitney test. The Kaplan-Meier method and the cumulative incidence function with correction for competing risks was used to estimate OS [15] and the log-rank test to examine the differences between curves. The expected years of life lost was calculated as the difference between the area under the survival curve of the reference population and the patient population [16]. The reference population was an age and sex matched Danish standard population [17]. A *p* < 0.05 was considered statistically significant. Statistical analysis was performed with GraphPad Prism software version 6.05. (GraphPad, La Jolla, CA).

## 3 Results

### 3.1 Patients

The study included 1,556 consecutive patients, 1,296 patients (83%) in group A and 260 patients (17%) in group B (Table 1). There were 1,208 patients with malignant tumors of whom 1,020 had adenocarcinoma. The remaining 348 patients had benign or no pathologic findings but were operated for pre-malignant diseases or on suspicion of a malignant tumor (Table 2).

**Table 1:** Clinical data of 1556 patients who underwent pancreatic surgery for benign and malignant disease.

	Total	<75 Years Benign	(group A) Malignant	≥75 Years Benign	(group B) Malignant
N	1556	300 (19%)	996 (64%)	48 (3%)	212 (14%)
Median age	66 (20 - 86)	64 (27–74)	65 (20–74)	77 (75 - 86)	78 (75 - 87)
Gender (M/F)	830/726 (53/47%)	152/148 (51/49%)	536/460 (54/46%)	28/20 (58/42%)	114/98 (64/46%)
Charlson Index					
0–1	1024 (66%)	261 (87%)	595 (60%)	44 (92%)	124 (59%)
2–3	411 (26%)	38 (13%)	303 (30%)	4 (8%)	66 (31%)
4–10	121 (8%)	1 (<1%)	98 (10%)	─	22 (10%)
Most common comorbidity					
Diabetes	258 (17%)	44 (15%)	167 (17%)	3 (6%)	44 (21%)
Heart disease and hypertension	549 (35%)	99 (33%)	337 (34%)	19 (40%)	94 (44%)
Pulmonary disease	83 (5%)	22 (7%)	45 (5%)	5 (10%)	11 (5%)
Renal disease	11 (1%)	3 (1%)	1 (<1%)	7 (15%)	─
Other	97 (6%)	20 (7%)	62 (6%)	2 (4%)	13 (6%)
Alcohol intake	493 (32%)	93 (31%)	308 (31%)	17 (75%)	75 (35%)
Tobacco	742 (48%)	130 (43%)	520 (52%)	9 (19%)	83 (39%)
Jaudice	609 (39%)	23 (8%)	476 (48%)	5 (10%)	105 (50%)
Preoperative biliary stent	528 (34%)	22 (7%)	411 (41%)	4 (8%)	91 (43%)

**Table 2:** Pancreaticoduodenal pathology of 1556 patients.

	Total	<75 years	≥75 years

Total	1556	1296	260

Malignant neoplasms			
Adenocarcinoma	1020	826	194
Pancreas	765	612	153
Periampullar	51	40	11
Distal bile duct	87	75	12
Duodenum	117	99	18

Neuroendocrine tumors (non-insulinoma)	110	100	10

Other malignant neoplasms	46	41	5
Metastases	32	29	3
Benign diseases			
Duodenal adenoma	48	43	5
IPMN	134	108	26
Cysts	47	41	6
Insulinoma	7	7	
Pancreatic dysplasia	10	10	
Pancreatitis and fibrosis	76	67	9
Other	15	15	
No pathology	11	9	2

Comorbidity was higher among patients with malignant diseases but independent of age group (*p* < 0.001). Heart disease and hypertension were more common in the elderly, while diabetes was more prevalent among patients with adenocarcinoma (*p* = 0.020). Regular intake of alcohol was unrelated to age group and adenocarcinomas, but tobacco smoking was more prevalent in patients with adenocarcinoma in pancreas and intrapancreatic bile duct (*p* < 0.001).

The distribution of pancreatic, papillary, intrapancreatic bile duct and duodenal adenocarcinoma was not significantly different between the two cohorts, nor was tumor stage and lymph node status (Table 3).

**Table 3:** Stage of adenocarcinomas from pathologic examination of resected specimens.

Years	Pancreas		Periampullar		Bile duct		Duodenum	
	<75	≥75	<75	≥75	<75	≥75	<75	≥75
N	612	153	40	11	75	12	99	18
I, IA IB	71	18	2	4	2	21	3	
IIA, IIB	291	78	28	1	49	10	23	4
III, IIIA, IIIB	247	56	10	6	23	55	11	
IV	3							
N0	243	49	17	5	23	5	44	7
N1	214	50	15	6	25	4	24	5
N2	155	54	8	27	3	31	6	

Nodes.

Pancreas, bile duct and periampullary: N0 no metastases, N1 1–3 metastases, N2 > 3 metastases.

Duodenum: N0 no metastases, N1 1–2 metastases, N2 > 2 metastases.

#### 3.1.1 Operations

The most common operation in both cohorts was pancreaticoduodenectomy followed by left pancreatectomy and total pancreatectomy (Table 4). Type of pancreatic resection was not significantly different between the cohorts, but porto-mesenteric venous resection was performed more frequent in patients younger than 75 years (*p* < 0.02). Distal pancreatectomy with celiac artery resection (DP-CAR, 21 patients) was only performed in patients younger than 75 years.

**Table 4:** Operations and major surgical complications.

	Total	<75 years	≥75 years

N	1556	1296	260
Pancreaticoduodenectomy	969 (62%)	810 (62%)	159 (60%)
Total pancreatectomy	278 (18%)	242 (19%)	36 (15%)
Left pancreatectomy	309 (20%)	244 (19%)	65 (25%)
Porto-mesenteric resection	230 (15%)	206 (16%)	24 (9%)
Arterial resection	36 (2%)	32 (3%)	4 (2%)
Major surgical complications (†deaths)			
Pancreatic fistula			
Pancreaticoduodenectomy	80 (8%)	67 (8%)	13 (8%)
Grade B	75	65	10
Grade C	8 (†4)	3 (†1)	5 (†3)
Left pancreatectomy	60 (17%)	49 (18%)	11 (17%)
Grade B	59	48	11
Grade C	1	1	
Bile fistula	80 (†4)	61 (†2)	19 (†2)
Hemorrhage	24 (†4)	21 (†3)	3 (†1)
Gastric bleeding	6	6	
Intestinal infarction	5 (†2)	4 (†2)	1
Intraabdominal abscess	29	26	3
Liver infarction	3 (†3)	3 (†3)	
Total	287 (19%)	237 (18%)	21 (19%)
Deaths	17 (1%)	11 (1%)	6 (2%)

Pancreatic fistula includes leakage after pancreaticoduodenectomy and left pancreatectomy. Bile fistula includes leakage from hepaticojejunostomy after pancreaticoduodenectomy and total pancreatectomy.

Twenty-nine patients, six in group B, were re-operated due to surgical complications, five of them with fatal outcome including three patients in group B. Five patients in group A were re-operated due to non-radicality evaluated from the final pathologic examination of the paraffin sections. Four of them had completion pancreatectomy with removal of the remaining pancreas, and one had re-resection of the pancreatic body after a distal pancreatectomy. All re-operations for non-radicality were performed without complications.

#### 3.1.2 Complications

Leakage from the pancreaticojejunostomy followed by leakage of the hepaticojejunostomy were the most common complications without a significant difference between the two cohorts. Type B pancreatic leakage and bile duct leakage were treated with antibiotics, ultrasound guided drainage and PTC assisted external bile drainage. Nine patients had a grade C pancreatic leakage, and four of them, all in group A, had a completion pancreatectomy. Other major complications included intraabdominal or gastric bleeding, intraabdominal abscess, liver infarction and necrosis of the transverse colon after total pancreatectomy (Table 4). Patients with gastric bleeding had gastroscopic hemostasis, while cases with intraabdominal bleeding were treated with a completion pancreatectomy (three patients) or were managed by radiologic intervention with coiling of the bleeding vessel (21 patients). One patient with intraabdominal abscess was operated, the remaining patients had ultrasonographic guided drainage. Four patients with liver infarction were all treated conservatively, three with lethal outcome.

#### 3.1.3 Postoperative mortality

The hospital stay was not significantly different in the two groups. The in-hospital mortality was 1.1% (group A) and 3.5% (group B), respectively, (*p* < 0.008). The mortality was related to surgical complications in 11 (0.9%) vs. four (1.5%) patients and to medical causes in 17 (1.3%) vs. seven (2.7%) patients, (Table 5). Most common death from medical cause in group A was heart conditions (12 patients) and respiratory failure (4 patients) in group B. There were no intra-operative deaths. The 30-days mortality was higher in group B (*P* < 0.041), but the 90-days mortality was not different in the two cohorts (*P* < 0.740) (Table 5).

**Table 5:** Hospital stay, postoperative mortality (all patients), and long-term mortality in patients with adenocarcinoma.

	<75 years	≥75 years	Total	

N	1296	260	1556	

Mortality until 90 days, all patients				
30 days	15 (1.2%)	8 (3.0%)	23 (1.5%)	*p* < 0.041
90 days	13 (1.0%)	3 (1.2%)	16 (1.0%)^a^	*p* = 0.740
In-hospital	14 (1.1%)	9 (3,5%)	23 (1.5%)	*p* < 0.008
Medical disorder	17 (1.3%)	7 (2.7%)	24 (1.5%)	*p* = 0.103
Surgical complication	11 (0.9%)	4 (1.5%)	15 (1.0%)	*p* = 0.296
Total	28 (2.2%)	11 (4.2%)	39 (2.5%)	*p* = 0.078
Hospital stay	12 (4 - 131)	11 (5 - 62)	12 (4 - 131)	*p* = 0.631
Mortality from 90 days, patients with adenocarcinoma				
N	826	194	1020	
Adenocarcinoma	433 (52.4%)	83 (42.8%)	516 (50.6%)	*p* = 0.017
Other cancer	11 (1.3%)	9 (4.6%)	20 (2.0%)	*p* = 0.066
Medical disorder	107 (13.0%)	33 (17.0%)	140 (13.7%)	*p* = 0.164
Total	551 (66.7%)	125 (64.4%)	676 (66.3%)	*p* = 0.555

a Six patients <75 years and one patient ≥75 years died from recurrence of malignant disease within three months from discharge.

#### 3.1.4 Follow-up and late outcome

The follow-up of patients with benign disease and adenocarcinoma was 57.95 (12.1–132.7) and 39.85 (12.0–131.7) months, respectively. The median OS of patients operated for non-malignant conditions was not reached, but the 5-year survival was 86.0% (group A) and 81.4% (group B), respectively (*p =* 0.3215).

**Fig. 1:**
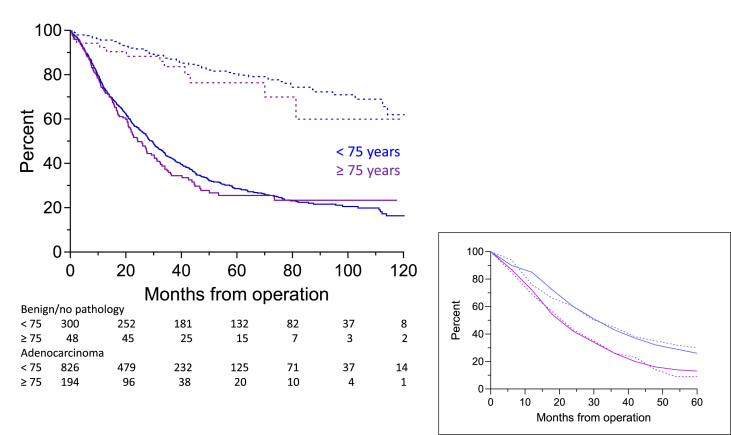
Postoperative overall survival of patients with benign disease/no pathology (dashed lines) and adenocarcinoma (solid lines). Inserted figure is the observed (solid lines) and predicted survival (dashed lines) of patients with adenocarcinoma. The curves are not significantly different (<75 years, *p* = 0.8438; ≥ *p* = 0.8955).

The predicted OS of patients with adenocarcinoma was 29.70 (group A) and 24.30 months (group B) (*p* = 0.3228) with a one, two, and five-year survival of 74.5%, 56.6% and 28.6% vs. 73.6%, 51.1%, and 25.5% (Fig. 1). The observed OS was 29.70 and 23.50 months, respectively (*p* = 0.1986) and not significantly different from the predicted survival.

**Fig. 2:**
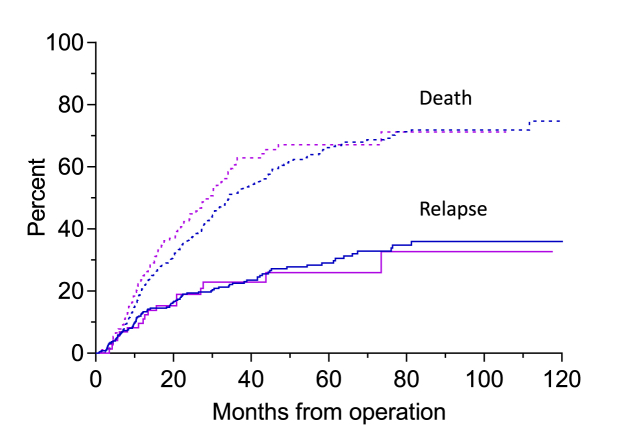
Cumulative incidence of death and relapse in patients with adenocarcinoma. The height of the lower curve is the cumulative incidence of relapse at time *t*. The distance between the top curve and the lower curve at is the cumulative incidence of the relapse-free mortality at time *t.* One minus the top curve is the cancer-free survival probability at *t* months after operation.

Relapse after adjustment for competing risks occurred in 46.2% of patients in group A and 40.5% in group B (*P* = 0.609), time to relapse was 9 (1 - 51) and 8 (1 - 78) months (p = 0.534), and the survival after relapse 21.0 (0.7–88.2) and 15.8 (1.6–63.2) months, respectively (p < 0.005) (Fig. 2).

**Fig. 3:**
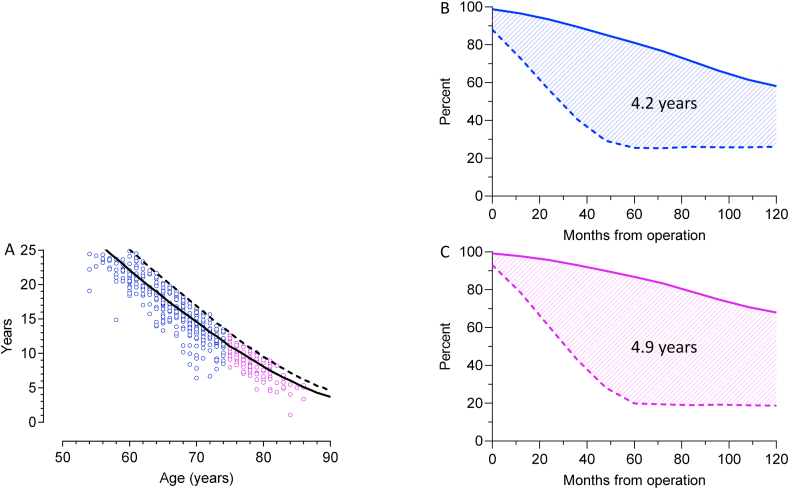
A. Scatterplots of the observed years of lost life. The lines are the expected remnant life of an age and sex matched Danish standard population, males: solid lines, females: dashed lines. Fig. 3B and C. The differences of life expectancy in patients ≥75 years with adenocarcinoma (dashed lines) and a sex- and age matched standard population (solid lines). The average expected years of lost lives in patients with adenocarcinoma is the difference between the area under the curves (AUC) of the reference groups and the survival curves of the patients. AUC males 4.2 years, females 4.9 years.

During follow-up, 551 patients with adenocarcinoma (66.7%) in group A and 125 patients (64.1%) in group B had died. Median time to death was 17.1 (0.1–113.8) and 15.6 (0.5–73.5) months, respectively. The observed years of lives lost was 16.6 (6.4–33.3) in group A and 8.7 (1.1–12.7) in group B equivalating 94% and 84%, respectively, of expected remnant life (*p* < 0.01) (Fig. 3 A). The average expected years of lives lost in the old cohort compared with a back-ground population was 4.2 years in male and 4.9 years in females (*p* = 0.025) (Fig. 3 B and C).

**Fig. 4:**
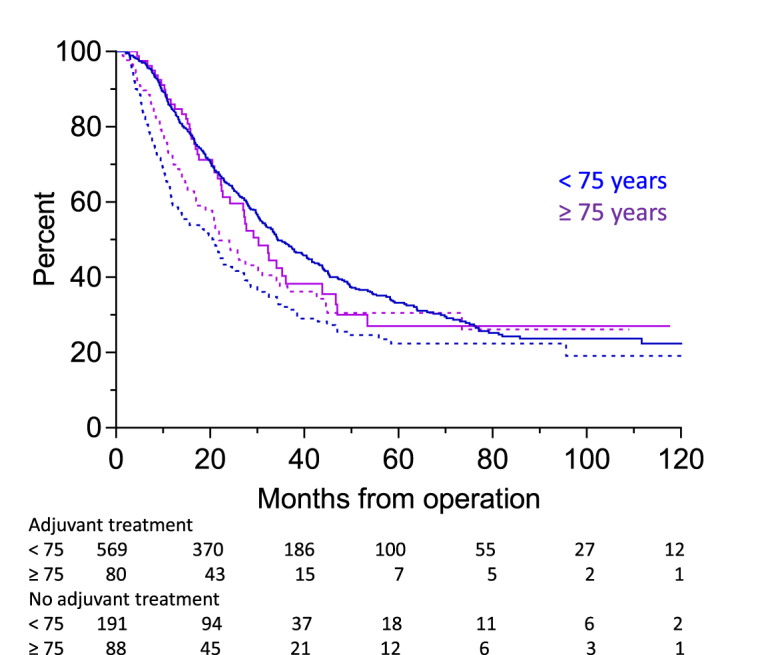
Postoperative cancer specific survival with (solid lines) and without adjuvant chemotherapy (dashed lines).

Adjuvant chemotherapy was completed in 72.0% of patients in group A and 45.1% of patients in group B (*p* < 0.0001). It had an impact on the outcome of patients in group A with a cancer specific survival of 40.9 (3.4–117.3) vs. 21.1 (3.1–103.2) months (*P* < 0.0001) in patients, who did not have adjuvant treatment. The survival of patients in group B with and without adjuvant therapy was 27.7 (4.9–81.7) and 21.1 (3.6–93.4) months, respectively (*P* = 0.475) (Fig. 4). When the results were evaluated in relation to stage, adjuvant chemotherapy was given more commonly in group B patients with N1 disease than in patients with N0 disease (*P* < 0.02), while a similar difference was not found among patients in the younger cohort (*P* = 0.129).

## 4 Discussion

The study showed that patients aged 75 years and older responded favorably to pancreatic surgery. The in-hospital mortality within first postoperative month was higher than in younger patients, but still within acceptable limits for large scale surgery, whereas hospital-stay and total number of complications were not different, neither did we find a higher number of surgical complications. Correction for the higher percentage of distal pancreatectomy, which is a lesser surgical intervention than a pancreaticoduodenectomy or a total pancreatectomy, did not influence the outcome.

The mortality during the first 90 postoperative days due to medical conditions were 61% and 63% of patients in group A and B, respectively. Different clinical parameters have been used to estimate the risk of pancreatic surgery, and here comorbidity comes in as the most important predictor for the postoperative survival [18]. However, patients who died in the present study did not have an inferior health status compared to those, who passed through the postoperative period. Nor was the mortality from medical disorders higher in the old cohort compared to patients younger than 75 years. The number of deaths from adenocarcinoma was higher in group A, probably due to the longer remnant life and thus higher risk to die from recurrence. However, the mortality due to other cancers was higher in group B, which is in accordance with the higher incidence of malignancies in elder patients.

There is a considerable variation in the reports on postoperative mortality not only internationally but also within countries, however, differences in stage, resection rate, patient volume and data retrieval make comparison of data difficult. The outcome of pancreatic surgery depends on the patient volume, and centralization of operations to high volume centers has a beneficial impact not only on mortality but also on the number of elder patients that are operated [[19], [20], [21]].

Results from single institutions vary with respect to postoperative mortality from population-based series. Single centers are often high-volume centers and present better results than population-based studies with different level institutions. Results from six centers in USA, Europe, and Asia each with more than 100 septuagenarians showed a postoperative mortality of 1.6%–12.9% [[22], [23], [24], [25], [26], [27]] and in octogenarians from six centers with 25 patients or more a mortality range between 0% and 5% [[28], [29], [30], [31], [32]]. These results and ours are in good agreement with The European Society of Medical Oncology (ESMO) guidelines that does not consider advanced age a contraindication for resection if comorbidity and functional status does not indicate otherwise.

When it comes to long-term survival after surgery for adenocarcinoma, the reported median survival after surgery for pancreatic adenocarcinoma has a wide range. For patients younger than 70 years it is 19–24 months, patients between 70 and 75 years 19–35 months and patients older than 75 years 15–30 months with an estimated five-year survival between 20 and 35% [20,22,26,27,33]. These figures are crude numbers as they do not differentiate between stage of disease or adjuvant oncologic therapy.

We found that elder patients received adjuvant chemotherapy or completed all cycles less often than patients in the younger cohort. Chemotherapy did not have the same impact on survival among the elderly although there was a longer but non-significant survival among those who received treatment. There could be several explanations, but a different selection of patients is probably the main reason for this paradox. Elder patients without lymph node metastases had less often adjuvant oncologic therapy than patients with lymph node involvement, i.e. patients under adjuvant oncologic therapy had a higher stage of disease, and thus a poorer prognosis. In addition, older patients are more likely to be given reduced doses of chemotherapy which may potentially compromise impact on the survival. From retrospective analysis of the ESPAC-3 trial the OS was better in patients who completed all six cycles of adjuvant chemotherapy versus those who did not [8]. Finally, the natural causes of death influence the survival in the old cohort.

Although the comorbidity among the elderly may exclude several of them from large scale surgery, it is often possible to improve their medical condition before surgery. Patients unfit for surgery or who abstain from surgical treatment may be offered palliative oncologic treatment, but palliative chemotherapy cannot offer patients the same long-term survival compared with radical surgery. Meta-analyses addressing older chemotherapy regimens for advanced pancreatic cancer have shown a significant survival benefit over best supportive care with an improved one-year mortality (odds ratio 0.37, 95% CI 0.25–0.57, one-year survival 58% versus 0%) [34,35]. Median OS of about 6, 8.5 and 11.1 months can be achieved with gemcitabine monotherapy, gemcitabine plus *nab*-paclitaxel and FOLFIRINOX, respectively [[36], [37], [38]]. Among these regimens the best one-year OS rate of 48.4% was reported in the FOLFIRINOX arm compared with 20.6% in the gemcitabine group. However, elderly patients are always underrepresented in large clinical trials. Patients older than 76 years were excluded from the FOLFIRINOX trial and only 10% of patients aged 75 years or older were enrolled in the randomized trial with gemcitabine and *nab*-paclitaxel. Patients older than 65 years appeared to achieve benefit from combination regimens, however, the degree of advantage was less prominent with higher age in a subset analysis [39,40].

The expected outcome after surgical treatment should exceed the outcome of palliative oncologic treatment or best supportive care. But the postoperative recovery and the patient's ability to live under changed circumstances such as enzyme substitution and insulin treatment needs evaluation. In the present study, patients who died from adenocarcinoma during the observation period, 66.7% in group A and 64.1% in group B, had a survival of 17.1 and 15.6 months, respectively, which equivalated to a loss of 94% and 86% of remnant life. Although almost two third of the patients died during the observation period, they still had a longer median survival than if treated with palliative chemotherapy. Moreover, old patients usually tolerate major surgery better than oncologic treatment.

Apart from operability, the surgeon should take the expected remaining life into account before surgery is considered an option. In 2019/2020 the life expectancy in Denmark was 81.5 years (males 79.5 and females 83.6 years). The expected remnant life of Danish males and females aged 75 and 86 years, which was the range of age of our old cohort was 10.6 and 12.5 vs. 5.0 and 6.2 years, respectively. Even with the average expected years of lives lost in the old cohort of 4.2 years in males and 4.9 years in females there was still a survival benefit from surgery in fit patients.

The population-based design from a single center with a follow-up based on a meticulous registration of health data is a major strength of the study. But it is relevant to point out that even though all patients had their operability evaluated before surgery, the number of patients who were denied operation or who made the decision themselves was higher in Group B, and thus the selection of patients is different in the two cohorts. This may to some extend influence the prediction of outcome parameters, but this bias should most likely be compensated by the large cohorts.

The population-based design from a single center with a follow-up based on a meticulous registration of health data is a major strength of the study. But it is relevant to point out that even though all patients had their operability evaluated before surgery, the number of patients who were denied operation or who made the decision themselves was higher in Group B, and thus the selection of patients is different in the two cohorts. This is a limitation of the study and may to some extend influence the prediction of outcome parameters, but this bias should most likely be compensated by the large cohorts.

## 5 Conclusion

In conclusion, our data support pancreatic surgery in elder patients both in term of postoperative mortality and long-term survival. Since ageing is an individual process, operability should be evaluated from morbidity and biological age and not from the chronological age alone. The prerequisite is that the incidence of severe postoperative complications is low and manageable, as old patients may not tolerate long hospital stay in intensive or high dependency units.

## Provenance and peer review

Not commissioned, externally peer-reviewed.

## Financial support

No funding

## Author contribution

SKB: Writing Original Draft, Writing Review & Editing
IMC: Writing Original Draft, Writing Review & Editing
JHS: Writing Original Draft, Writing Review & Editing
CPH: Conceptualization, Data Curation, Methodology, Formal Analysis, Writing Original Draft, Writing Review & Editing,

## Research Registration Unique Identifying Number (UIN)

Name of the registry: clinicaltrials.gov
Unique Identifying number or registration ID: NCT04893408
Hyperlink to your specific registration (must be publicly accessible and will be checked): https://clinicaltrials.gov/ct2/show/NCT04893408?term=NCT04893408&draw=2&rank=1.

## Guarantor

Stefan Kobbelgaard Burgdorf

## Declaration of competing interest

Dr. Burgdorf has nothing to disclose.

Dr. Storkholm has nothing to disclose.

Dr. Chen has nothing to disclose.

Dr. Palnæs has nothing to disclose.
